# Five‐year cumulative incidence of overweight and obesity, and longitudinal change in body mass index in Japanese workers: The Japan Epidemiology Collaboration on Occupational Health Study

**DOI:** 10.1002/1348-9585.12095

**Published:** 2019-11-02

**Authors:** Miyuki Hasegawa, Shamima Akter, Huanhuan Hu, Ikuko Kashino, Keisuke Kuwahara, Hiroko Okazaki, Naoko Sasaki, Takayuki Ogasawara, Masafumi Eguchi, Takeshi Kochi, Toshiaki Miyamoto, Tohru Nakagawa, Toru Honda, Shuichiro Yamamoto, Taizo Murakami, Makiko Shimizu, Akihiko Uehara, Makoto Yamamoto, Teppei Imai, Akiko Nishihara, Kentaro Tomita, Satsue Nagahama, Ai Hori, Maki Konishi, Isamu Kabe, Tetsuya Mizoue, Naoki Kunugita, Seitaro Dohi, the Japan Epidemiology Collaboration on Occupational Health Study Group, T. Mizoue, S. Akter, H. Hu, Y. Inoue, A. Fukunaga, I. Kashino, Z. Islam, M. Konishi, A. Nanri, K. Kurotani, K. Kuwahara, T. Nakagawa, S. Yamamoto, T. Honda, Y. Watanabe, S. Dohi, H. Okazaki, T. Imai, A. Nishihara, N. Sasaki, T. Ogasawara, A. Uehara, M. Yamamoto, T. Miyamoto, M. Hasegawa, M. Shirozu, I. Kabe, T. Kochi, M. Eguchi, T. Murakami, C. Shimizu, M. Shimizu, N. Gonmori, A. Ogasawara, N. Kato, A. Tomizawa, K. Tomita, S. Nagahama, N. Kunugita, T. Sone, K. Fukasawa, A. Hori, C. Nishiura, C. Kinugawa, R. Kuroda, K. Yamamoto, M. Ohtsu, N. Sakamoto, Y. Osaki, T. Totsuzaki, M. Endo, T. Itoh, M. Kawashima, M. Masuda, K. Kitahara, T. Yokoya, K. Fukai, K. Odagami, Y. Kobayashi

**Affiliations:** ^1^ Department of Epidemiology and Prevention Center for Clinical Sciences National Center for Global Health and Medicine Tokyo Japan; ^2^ Teikyo University Graduate School of Public Health Tokyo Japan; ^3^ Mitsui Chemicals, Inc Tokyo Japan; ^4^ Mitsubishi Fuso Truck and Bus Corporation Kanagawa Japan; ^5^ Furukawa Electric Co., Ltd Tokyo Japan; ^6^ Nippon Steel Corporation Chiba Japan; ^7^ Hitachi, Ltd Ibaraki Japan; ^8^ Mizue Medical Clinic Keihin Occupational Health Center Kanagawa Japan; ^9^ Seijinkai Shizunai Hospital Hokkaido Japan; ^10^ Yamaha Corporation Shizuoka Japan; ^11^ Azbil Corporation Tokyo Japan; ^12^ Healthplant Tokyo Japan; ^13^ All Japan Labour Welfare Foundation Tokyo Japan; ^14^ Department of Global Public Health University of Tsukuba Ibaraki Japan; ^15^ National Institute of Public Health Saitama Japan

**Keywords:** body mass index, epidemiology, Japanese worker, obesity, overweight

## Abstract

**Objective:**

The present study aimed to estimate cumulative incidence of overweight and obesity and describe 5‐year longitudinal changes in body mass index (BMI) in a large occupational cohort in Japan.

**Methods:**

Participants were 55 229 Japanese employees, who were aged 20‐59 years and attended at all subsequent annual health check‐ups between 2009 and 2014. Mixed model analysis was performed to examine the effects of age and cohort by gender on BMI change, with age as a random variable. Cumulative incidence of overweight (23.0≤ BMI <27.5 kg/m^2^) and obesity (BMI ≥27.5 kg/m^2^) was calculated. Logistic regression analysis was used to estimate odds ratios for the incidence of overweight and obesity according to age group.

**Results:**

The incidence of overweight and obesity was approximately double in men (28.3% and 6.7%, respectively) compared to women (14.3% and 3.9%, respectively).The incidence of obesity decreased with age in men, but did not differ according to age in women (*P* for trend: .02 and .89, respectively). Among overweight participants, the incidence of obesity was higher in women (18.9%) than men (14.5%) and decreased with advancing age (*P* for trend: <.001 in men and .003 in women). Mean BMI was higher in men than women in all age groups throughout the period. Younger cohorts tended to have a higher BMI change compared with older cohorts.

**Conclusions:**

In this Japanese occupational cohort, transition from overweight to obesity is higher in women than men, and the more recent cohorts had a higher change in mean BMI than the older cohorts.

## 1 INTRODUCTION

Obesity is a global epidemic.^1^ It has been estimated that the worldwide prevalence of overweight or obesity has increased from 28.8% to 36.9% in men and 29.8% to 38.0% in women between 1980 and 2013.^2^ In Japan, the prevalence of overweight and obesity has remained largely stable in women, but has increased 1.5‐fold in men over the past three decades; the change is striking in men in their 20s, for whom obesity levels have approximately doubled.^3^, ^4^ Overweight and obesity are risk factors for non‐communicable diseases including cancer, cardiovascular disease and type 2 diabetes.^5^ Thus, appropriate weight control is crucial in the prevention of non‐communicable disease.

At national level, the trend of obesity over time has been described based on repeated cross‐sectional surveys.^6^, ^7^ To formulate effective strategies for non‐communicable disease prevention, it is important to identify groups with high risk of developing obesity using a longitudinal design. Several studies have shown longitudinal data on obesity among adults in Western countries,^8^, ^9^, ^10^, ^11^, ^12^ the Middle East,^13^ the West Indies^14^ and China.^15^ In Japan, a large, community‐based prospective study presented a 10‐year change in overweight and obesity,^16^ based on self‐reported anthropometric data. However, such data on obesity is lacking among working population. The risk of obesity must be much higher among overweight individuals than those with normal weight, but quantitative data on transition from overweight to obesity is lacking among Japanese. Such data would be useful to reduce obesity by implementing effective prevention strategies such as early treatment and lifestyle modification among the target population (overweight). In Japan, national health policy has highlighted the need to reduce prevalence of overweight and obesity, but the goal has not been achieved yet.^17^ Worksite weight control program targeting for workers may help to accomplish the goal.

The aim of this study was to estimate the cumulative incidence of overweight and obesity amongst a working population over a 5‐year follow‐up period between 2009 and 2014 and to describe longitudinal change in BMI, using data from a large‐scale multi‐company cohort study in Japan.

## 2 METHODS

### 2.1 Survey setting

The Japan Epidemiology Collaboration on Occupational Health (J‐ECOH) study is an ongoing multi‐center epidemiological study among Japanese workers from more than twelve companies covering various industries(electric machinery and apparatus manufacturing; steel, chemical, gas and non‐ferrous metal manufacturing; automobile and instrument manufacturing; plastic product manufacturing; and healthcare).These companies are mainly located in semi‐urban and urban areas of Kanto and Tokai region of Japan; however, branch office in some companies are located throughout Japan. As of May 2012, twelve companies participated in the study and eleven provided data on periodic health check‐up. The present study is based on health check‐up data collected between January 2009 and December 2014, or between April 2009 and March 2015. The details of the J‐ECOH study have been described elsewhere.^18^, ^19^ In Japan, employees are obliged to undergo health examinations at least once a year under the Industrial Safety and Health Act.^20^ The J‐ECOH study was announced in each participating company through a poster; workers did not provide oral or written informed consent but were given the opportunity to opt for non‐participation in this study, in accordance with the Japanese Ethical Guidelines for Epidemiological Research.^21^ The study protocol was approved by the Ethics Committee of the National Center for Global Health and Medicine, Japan.

### 2.2 Study population

The present analysis was done using data of those who attended all the six health check‐ups from fiscal 2009 (baseline) through fiscal 2014. Of the 82 803 participants who were aged 20‐59 years and had anthropometric measurements taken in 2009, we excluded those who had missing data for gender, age, worksite, current status of smoking (n = 4138), and those who did not attend all subsequent anthropometric measurements (n = 22 557). Of the remaining 56 108 participants, we further excluded those who had a self‐reported medical history of cancer (data available in ten companies) and stroke, myocardial infarction or angina pectoris (data available in eight companies) (n = 879), leaving 55 229 participants (48 432 men and 6797 women) for the analysis. Compared to those who were included in the present study (n = 55 229), those who were excluded due to missing anthropometric data for all subsequent health checkups (n = 22 557) were older (mean age, 44.5 years vs. 40.9 years), tended to be women (21.9% vs. 12.3%) and nonsmoker (33.9% vs. 39.2%) and had a lower BMI (mean BMI, 23.1 kg/m^2^ vs. 23.3 kg/m^2^).

### 2.3 Outcomes

Body weight and body height were measured while participants were wearing light clothing and no shoes. BMI was calculated by dividing body weight in kilograms with squared body height in meters. Two sets of BMI cut‐off points were used to determine whether participants were overweight or obese: the WHO recommendation for Asian populations (normal weight: <23.0 kg/m^2^, overweight: ≥23.0 kg/m^2^ to <27.5 kg/m^2^; obese: ≥27.5 kg/m^2^),^22^ and WHO classification (normal weight: <25.0 kg/m^2^, overweight: ≥25.0 kg/m^2^ to <30.0 kg/m^2^; obese: ≥30.0 kg/m^2^).^23^

### 2.4 Other variables

Medical history and smoking habit (current or non‐current) were identified via a self‐administered questionnaire. We adjusted for smoking in the models because smoking status is associated with BMI^24^ and this information is available across the participating companies. We also adjusted for worksite (eleven worksites) because background characteristics and BMI might differ in different companies.

### 2.5 Statistical analysis

Descriptive statistics were expressed using percentage and mean value according to gender and 5‐year age groups. Mixed model analysis was performed to examine the effects of age and cohort on annual BMI change during 5‐year follow‐up by gender, with age as a random variable. Two different models were fitted. The first model was unadjusted, and the second model was adjusted for smoking (current or non‐current) and worksites (eleven worksites). Models were fitted with fixed and random individual‐level effects and random slopes was used to assess differences in BMI within individuals over time (age effect) and to assess differences in experienced time effect across individuals of varying ages (cohort effect). Unstructured covariance was considered that allows for all variances and covariances to be distinct. The coefficients for age can be interpreted as overall age effects after controlling for other factors. The coefficients for time and age interaction terms can be interpreted as differences in experienced time effect across individuals of varying ages (cohort effect). Smoking and worksites were entered as fixed variables in the model. Because we are using panel data and smoking status has repeated measurements at each follow‐up, the modeling will consider change in smoking status during follow‐up.

The cumulative incidence of overweight was the proportion of new‐onset cases of overweight that developed during the follow‐up period from normal weight participants at baseline. The cumulative incidence of obesity was the proportion of new‐onset cases of obesity that developed during the follow‐up period from non‐obese participants at baseline. The incidence of overweight and obesity were calculated according to gender, age group and BMI classification at baseline. Logistic regression analysis was performed to estimate the odds ratio (OR) and 95% confidence interval (CI) for the incidence of overweight and obesity according to age group, adjusted by worksite (eleven worksites) and smoking status(current or non‐current) at baseline, and using 50‐54 years as the reference age group. An alternative approach using Cox proportional hazards regression considering longitudinal data who had baseline health checkup data and at least one subsequent health checkup data (n = 73 752; 63 093 men and 6797 women) were used to assess incidence rate and hazard ratio of overweight and obesity according to baseline age groups.

Tests for trend were carried out by assigning ordinal numbers to each of the age categories and modeling these as continuous variables. Two‐sided *P*‐values <0.05 were regarded as significant. All statistical analysis was performed by SAS (ver. 9.3, SAS Institute) or Stata (MP Version 15.1; Stata Corporation).

## 3 RESULTS

Table 1 shows the baseline characteristics of the study population according to age group. Among the total study population, the mean BMI was 23.5 kg/m^2^ in men and 21.6 kg/m^2^ in women. The prevalence of overweight and obesity were 40.3% and 11.2% in men, and 18.6% and 7.1% in women, respectively, by the BMI cut‐off points for Asians. The proportions of current smokers were 43.4% for men and 11.5% for women. The prevalence of overweight appeared to increase steadily with age in both genders. The prevalence of obesity was higher in participants aged 35‐49 years for men and in those aged 45‐59 years for women, compared with other age groups.

**Table 1:** Baseline characteristics of study population by age group

	Age group (years)								Total
	20‐24	25‐29	30‐34	35‐39	40‐44	45‐49	50‐54	55‐59	
Men									
No.	2600	4292	5593	9162	8755	7533	6913	3584	48 432
BMI (kg/m^2^)	22.3 ± 3.7	22.8 ± 3.6	23.2 ± 3.6	23.7 ± 3.6	23.8 ± 3.4	23.9 ± 3.2	23.7 ± 3.0	23.7 ± 2.9	23.5 ± 3.4
Overweight (%)^a^	24.5	30.3	34.9	39.4	42.4	44.3	46.9	48.2	40.3
Overweight (%)^b^	12.3	15.5	19.9	23.5	26.2	26.5	26.7	27.0	23.4
Obesity (%)^a^	7.9	9.0	10.8	12.2	13.0	12.3	10.4	8.8	11.2
Obesity (%)^b^	4.5	4.4	4.9	5.2	4.9	4.5	3.0	2.3	4.4
Current smoker (%)	44.0	42.8	46.8	45.9	41.7	42.0	42.0	41.2	43.4
Women									
No.	381	510	726	1614	1353	1041	808	364	6797
BMI (kg/m^2^)	21.0 ± 3.2	20.7 ± 3.2	21.3 ± 3.6	21.2 ± 3.6	21.6 ± 3.5	22.0 ± 3.7	22.2 ± 3.6	22.5 ± 3.5	21.6 ± 3.6
Overweight (%)^a^	13.9	10.2	16.1	15.6	19.4	20.6	25.5	30.5	18.6
Overweight (%)^b^	7.6	6.9	9.8	9.1	10.8	12.6	14.1	21.4	11.1
Obesity (%)^a^	5.0	5.9	6.8	5.9	6.7	9.3	8.4	9.3	7.1
Obesity (%)^b^	2.4	2.2	3.0	3.2	3.5	3.9	3.7	1.4	3.2
Current smoker (%)	10.8	11.8	16.4	12.2	10.5	10.3	9.2	11.0	11.5

Note

Data were expressed as mean ± SD or as percentage.

Abbreviation: BMI, body mass index.

a BMI cut‐off points for Asian populations: overweight: 23.0 kg/m^2^ ≤ BMI<27.5 kg/m^2^, obesity: ≥27.5 kg/m^2^.

b WHO BMI classification: overweight: 25.0 kg/m^2^ ≤ BMI<30.0 kg/m^2^, obesity: BMI ≥ 30.0 kg/m^2^_._

Table 2 shows the 5‐year cumulative incidence of overweight and obesity, and the adjusted ORs for the incidence according to age group. The crude incidence of overweight was 28.3% and 14.3% in men and women, respectively. The crude incidence of obesity was 6.7% and 3.9% in men and women, respectively. Men had a greater incidence of overweight and obesity across all age groups compared to women. The adjusted ORs for the incidence of overweight were slightly higher (in the range of less than 15%) among the participants aged 35‐44 years than other age groups in men, and the *P*‐values for trend (age groups) were non‐significant in both genders (*P* for trend: .313 for men and .557 for women). The adjusted ORs for the incidence of obesity tended to decrease with advancing age in men but not in women (*P* for trend: .018 for men and .892 for women).A similar trend of association was found using Cox proportional hazards model (Table S1). Based on the BMI cut‐off points by WHO classification,^22^ the incidence of obesity was 2.9% and 2.3% in men and women respectively (Table S2).

**Table 2:** Cumulative incidence of overweight and obesity by age group during 5‐year follow‐up period

Age group (years)	Men			Women		
	No.	Incidence, no. (%)	OR (95% CI)^c^	No.	Incidence, no. (%)	OR (95% CI)^c^
Overweight^a^						
Total	23 511	6648 (28.3)	—	5049	723 (14.3)	—
20‐24	1758	492 (28.0)	1.02 (0.89‐1.16)	309	49 (15.9)	1.15 (0.77‐1.70)
25‐29	2605	733 (28.1)	1.05 (0.94‐1.19)	428	44 (10.3)	0.70 (0.47‐1.04)
30‐34	3038	842 (27.7)	1.03 (0.92‐1.15)	560	90 (16.1)	1.17 (0.84‐1.65)
35‐39	4436	1304 (29.4)	1.12 (1.01‐1.24)	1268	181 (14.3)	1.04 (0.78‐1.40)
40‐44	3904	1140 (29.2)	1.11 (1.00‐1.24)	1001	156 (15.6)	1.16 (0.86‐1.57)
45‐49	3274	934 (28.5)	1.07 (0.96‐1.19)	730	105 (14.4)	1.04 (0.75‐1.44)
50‐54	2955	811 (27.4)	1.00 (reference)	534	73 (13.7)	1.00 (reference)
55‐59	1541	392 (25.4)	0.90 (0.78‐1.03)	219	25 (11.4)	0.77 (0.47‐1.26)
*P* for trend	—	—	.313	—	—	.577
Obesity^b^						
Total	43 032	2875(6.7)	—	6315	248 (3.9)	—
20‐24	2394	179 (7.5)	1.34 (1.11‐1.61)	362	15 (4.1)	1.20 (0.62‐2.33)
25‐29	3906	279 (7.1)	1.31 (1.11‐1.54)	480	16 (3.3)	0.95 (0.50‐1.82)
30‐34	4990	328 (6.6)	1.19 (1.02‐1.39)	677	31 (4.6)	1.28 (0.74‐2.21)
35‐39	8047	584 (7.3)	1.33 (1.16‐1.52)	1519	64 (4.2)	1.24 (0.77‐2.00)
40‐44	7620	556 (7.3)	1.35 (1.17‐1.55)	1263	53 (4.2)	1.25 (0.76‐2.05)
45‐49	6610	418 (6.3)	1.15 (0.99‐1.33)	944	29 (3.1)	0.89 (0.51‐1.55)
50‐54	6195	347(5.6)	1.00 (reference)	740	24 (3.2)	1.00 (reference)
55‐59	3270	184 (5.6)	1.00 (0.83‐1.20)	330	16 (4.8)	1.38 (0.72‐2.65)
*P* for trend	—	—	.018	—	—	.892

Note

BMI cut‐off points for Asian populations: overweight: 23.0 kg/m^2^ ≤ BMI<27.5 kg/m^2^; obesity: ≥27.5 kg/m^2^.

a Number of normal weight participants at baseline.

b Number of non‐obese participants at baseline.

c Based on logistic regression analysis. Odds ratio (OR) with 95% confidence interval (CI) was adjusted by worksite (11 work site) and smoking status (current or non‐current) at baseline.

Table 3 shows the 5‐year cumulative incidence of obesity and the adjusted ORs for the incidence according to age group among overweight participants. Among those who were overweight at baseline, the incidence of obesity was slightly higher in women (18.9%) than in men (14.5%) and decreased with advancing age in both genders. Similarly, the adjusted ORs decreased with advancing age (*P* for trend: <.001 for men and .003 for women), with the highest ORs of 3.02 (95%CI: 2.45‐3.73) for men in their early 20s and 3.01 (1.43‐6.36) for women in their late 20s.). A similar trend of association was found using Cox proportional hazards model (Table S3). On the other hand, among participants with normal weight at baseline, the incidence of obesity was far lower at 0.2% in both genders (data not shown).

**Table 3:** Cumulative incidence of obesity in overweight participants by age group during 5‐year follow‐up period

Age group (years)	Men			Women		
	No.	Incidence, no. (%)	OR (95% CI)^a^	No.	Incidence, no. (%)	OR (95% CI)^a^
Total	19 521	2828 (14.5)	—	1266	239 (18.9)	—
20‐24	636	170 (26.7)	3.02 (2.45‐3.73)	53	14 (26.4)	2.54 (1.18‐5.46)
25‐29	1301	264 (20.3)	2.12 (1.78‐2.52)	52	15 (28.8)	3.01 (1.43‐6.36)
30‐34	1952	325 (16.6)	1.66 (1.41‐1.95)	117	29 (24.8)	2.55 (1.38‐4.71)
35‐39	3611	576 (16.0)	1.59 (1.38‐1.84)	251	62 (24.7)	2.53 (1.50‐4.28)
40‐44	3716	548 (14.7)	1.47 (1.27‐1.70)	262	52 (19.8)	1.86 (1.09‐3.18)
45‐49	3336	416 (12.5)	1.21 (1.04‐1.40)	214	28 (13.1)	1.15 (0.64‐2.08)
50‐54	3240	345 (10.6)	1.00 (reference)	206	23 (11.2)	1.00 (reference)
55‐59	1729	184 (10.6)	1.01 (0.84‐1.22)	111	16 (14.4)	1.29 (0.65‐2.57)
*P* for trend	—	—	<.001	—	—	.003

Note

BMI cut‐off points for Asian populations; overweight: 23.0 kg/m^2^ ≤ BMI<27.5 kg/m^2^, obesity: ≥27.5 kg/m^2^.

a Based on logistic regression analysis. Odds ratio (OR) with 95% confidence interval (CI) was adjusted by worksite (11 work site) and smoking status (current or non‐current) at baseline.

**Figure 1:**
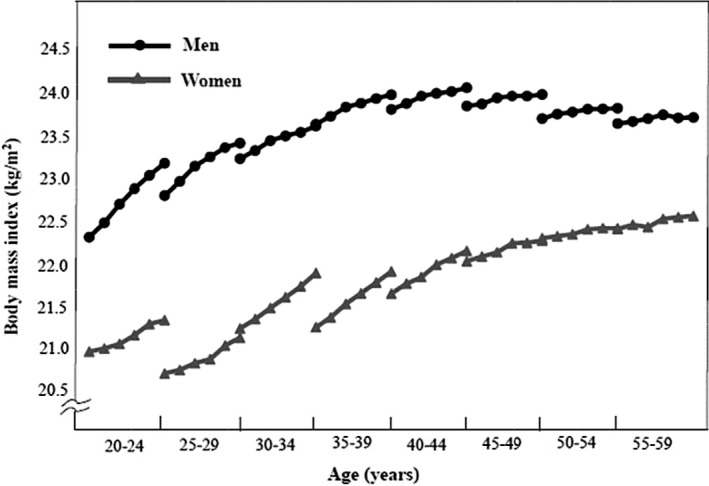
Changes in mean body mass index by age and birth cohort group during 5‐year follow‐up period among men and women. Estimations are based on unadjusted linear mixed effects model. Points represent mean BMI (kg/m^2^) by baseline age groups for each cohort across time (2009, 2010, 2011, 2012, 2013, 2014)

Table 4 shows age, time, and cohort effects on mean BMI change by gender. A positive cohort effect was observed in both genders with more recent cohorts having higher BMI relative to the referent cohort (year 2009). Figure 1 shows the change in mean BMI during the 5‐year follow‐up according to age and birth cohort by gender. Mean BMI values of the participants according to gender and 5‐year age groups were estimated at baseline and at each 5 consecutive follow‐ups. Mean BMI was higher in men than women in all age groups throughout the period. There was a significant cohort effect; more recent cohorts had a higher increase in mean BMI compared with older cohorts in both men and women (*P* < 0.001). The greatest annual changes in BMI occurred in men aged 20‐24 years and in women aged 30‐39 years among all cohorts. In women, average BMI was<23 kg/m^2^ across all age and birth cohorts and younger women had lower mean BMI than older women.

**Table 4:** Parameter estimates (95% confidence intervals) from linear mixed effects models predicting BMI (kg/m^2^) among working population

	Men		Women	
	Model 1	Model 2^b^	Model 1	Model 2^b^
Age (years)	0.029 (0.020, 0.033)	0.033 (0.027, 0.031)	0.065 (0.054, 0.076)	0.068 (0.057, 0.079)
Time (survey year)^a^				
2010	0.211 (0.172, 0.249)	0.200 (0.161, 0.239)	0.087 (−0.017, 0.191)	0.076 (−0.028, 0.181)
2011	0.486 (0.445, 0.527)	0.473 (0.432, 0.514)	0.272 (0.160, 0.385)	0.266 (0.153, 0.379)
2012	0.631 (0.586, 0.676)	0.623 (0.578, 0.669)	0.357 (0.230, 0.483)	0.337 (0.211, 0.464)
2013	0.833 (0.783, 0.883)	0.821 (0.771, 0.871)	0.621 (0.48, 0.76)	0.594 (0.450, 0.737)
2014	0.972 (0.916, 1.028)	0.950 (0.894, 1.006)	0.808 (0.645, 0.972)	0.768 (0.603, 0.932)
Time × Age				
2010 × Age	−0.0038 (−0.0047, −0.0029)	−0.0038 (−0.0047, −0029)	−0.0017 (−0.0042, 0.0007)	−0.0015 (−0.0040, 0.0010)
2011 × Age	−0.0086 (−0.0096, −0.0077)	−0.0086 (−0.0096, −0.0076)	−0.0056 (−0.0082, −0.0030)	−0.0056 (−0.0082, −0.0030)
2012 × Age	−0.0113 (−0.0122, −0.0102)	−0.0113 (−0.0123, −0.0103)	−0.0063 (−0.0092, −0.0035)	−0.0061 (−0.0090, −0.0033)
2013 × Age	−0.0153 (−0.0164, −0.0142)	−0.0153 (−0.0164, −0.0142)	−0.0117 (−0.0148, −0.0085)	−0.0113 (−0.0144, −0.0081)
2014 × Age	−0.0177 (−0.0189, −0.0165)	−0.0179 (−0.0191, −0.0167)	−0.0153 (−0.0187, −0.0118)	−0.0149 (−0.0184, −0.0114)
Smoking (Current smoker)^a^		−0.304 (−0.319, −0.288)		0.134 (−0.200, −0.068)

a Year 2009 and non‐current smoker is considered as referent group in analysis.

b Model 2 was adjusted for worksite (11 work site) and smoking status (current or non‐current).

## 4 DISCUSSION

In this study, we described longitudinal changes in BMI and estimated the 5‐year incidence of overweight and obesity in a large Japanese working cohort. During the follow‐up period, the BMI increased consistently with age across all birth cohorts in both genders, and younger cohorts had higher increase in mean BMI compared with older cohorts. The incidence of overweight was 28.3% and 14.3% for all men and women, respectively, and did not vary significantly by age groups. The incidence of obesity was 6.7% and 3.9% for all men and women, respectively, and showed a decreasing trend with advancing age in men but not in women. Among those who were overweight, the incidence of obesity was slightly higher in women than in men, and decreased significantly with advancing age in both genders.

The present study found a higher change in mean BMI among younger cohorts in both men and women. In a Japanese study,^25^ which was based on the repeated cross‐sectional National Nutritional Survey in Japan (NNS‐J) between 1956 and 2005 in community populations (aged 20‐69 years) reported a greater increase in BMI in more recent birth cohorts in men but not in women. Another longitudinal study^16^ based on self‐reported BMI recording the 10‐year change in BMI in Japanese community population (aged ≥40 years) also showed a higher change in mean BMI among younger cohorts in men but not in women. The findings from these previous studies were not directly comparable with the present study given the different study population (working vs. community population), study design (longitudinal vs. cross‐sectional) and age groups (≥20 years vs. ≥40 years). The findings of our study are in line with a Chinese longitudinal study,^26^ where younger cohorts had a higher mean BMI irrespective of gender. The findings of our study are also in line with previous findings from Western countries. Several longitudinal studies from United States (US),^27^ Norway,^28^ Sweden^29^ and Netherlands,^30^ reported a larger increase in BMI in younger cohorts in both men and women. The greater increase in BMI in younger cohorts than older cohorts could be ascribed to the difference of lifestyles between them. Globally, the consumption of sugar‐sweetened beverages is higher in younger cohorts.^31^ In Japan, the consumption of animal protein and fat is increasing but daily physical activity is decreasing in younger cohorts.^32^ In addition, younger cohorts who just started to work after completing graduation from schools might have higher stress that may influence their BMI levels.^33^ Given the potential threat of obesity to public health, it is important to find out the reasons for the obesity epidemic in younger cohorts so that effective interventions can be implemented.

In the present study, men had a twofold higher incidence of overweight and obesity compared with women. In keeping with our result, a Japanese study amongst community populations (aged 40‐69 years) reported a higher incidence of overweight and obesity in men than in women.^16^ In the Framingham Heart Study, in which participants were aged 30‐59 years,^11^ the 4‐year rates of becoming overweight were 26%‐30% in men and 14%‐19% in women, and those of developing obesity were 7%‐9% in men and 5%‐7% in women (for age groups 30‐39, 40‐49 and 50‐59 years).In a Greek study among community residents (aged ≥20 years), the 5‐year incidence of obesity was twice as high in men (21.8%) than in women (11.9%).^8^ On the other hand, an Australian study showed no gender difference in the incidence of overweight and obesity,^12^ whilst an Iranian study reported a higher incidence of these conditions in women than in men.^13^ It is difficult to compare gender difference in the incidence among the countries due to the differences in age and the definition of overweight and obesity. The reasons for the higher incidence of overweight and obesity in men than women in the present study is not clear but may be due to gender differences in social, behavioral and biological factors. Specifically, diet, physical activity, smoking and alcohol drinking, all of which are known to be associated with weight changes,^34^, ^35^, ^36^ differ much between men and women in Japan.^4^, ^37^ Sex hormones, which regulate lipid and lipoprotein metabolism,^38^ and sex‐specific perceptions for body shape^39^ can also contribute to the variations in the pattern of weight gain between men and women.

As regard progression from overweight to obesity, we found that the incidence of obesity among overweight participants was slightly higher in women (18.9%) than in men (14.5%), and decreased with advancing age in both genders. In a Spanish study,^9^ the incidence of obesity among overweight individuals was twice as high, or greater, in women (29.9%) than in men (13.1%). In a US study,^11^ the incidence of obesity among overweight group was also higher in women (16%‐23%) than in men (12%‐13%) across all age groups, and appeared to decline with advancing age in women but not in men. An African study found the 9‐year rate of developing obesity among overweight individuals was higher in women (19.9%‐28.6%) than in men (10.7%‐19.4%) across all age groups (40 years or older), and showed a decreasing trend with advancing age among black populations in both genders.^14^ In contrast, the Greek study^8^ reported no gender difference in the incidence of obesity among overweight participants. It is not clear why incidence of obesity is higher among overweight women than overweight men. One reason may be that excess body fat leads to hormonal imbalance and reproductive disturbances throughout women's lives^40^ and that fluctuations in reproductive hormone concentrations among overweight women uniquely predispose them to excess weight gain.^41^ Another reason may be that men who had muscularity body can be recognized as overweight although they have healthful levels of fat and their progression from overweight to obesity is less common. However, given that adults with high BMI are likely to have high proportion of body fat,^42^ this possibility would be low in the current working population.

The main strengths of this study include its prospective design, large sample size and objective measurement of BMI. In addition, weight was measured annually during follow‐up, which allowed us to capture the incidence of overweight and obesity more intensively. However, several limitations need to be considered. First, information on serious illness was not available in a few of the participating companies. We observed, however, a similar incidence rate of overweight and obesity after excluding subjects with history of cancer and cardiovascular disease, for which information was available in 8 companies. Second, the sample size for women was relatively small so that the estimates obtained were unstable. Third, we did not collect data on diet or lifestyle factors (eg alcohol consumption, physical activity, stress, living alone or with family, and socio‐economic status etc) except for smoking in a standardized manner across the participating companies and thus, we were unable to adjusted for these covariates. Fourth, for women, we have no information and data regarding the pregnancy status and thus, we were unable to exclude pregnant women from our analysis that might bias the estimation of obesity and weight change. Fifth, participants who were excluded from the present analysis for not attending any subsequent health checkups were older, tended to be nonsmoker and had lower BMI. Inclusion of such selective population might bias the estimation and association of overweight/obesity according to age groups. Sixth, our study subjects were employees mainly of large manufacturing companies in Japan. Therefore, it may not be possible to generalize the findings to other populations, such as those working for small‐ and middle‐scale companies and those with different occupational backgrounds.

## 5 CONCLUSIONS

In this Japanese occupational cohort, BMI increased consistently with age across all cohorts in both genders. The more recent cohorts had a higher increase in mean BMI than the older cohorts. The 5‐year incidence of overweight and obesity were approximately double in men compared to women. Among the overweight participants, the incidence of obesity was slightly greater in women than in men and tended to decline significantly with advancing age in both genders. The findings of our study emphasized the need for workplace obesity prevention program, especially for young men, in Japan.

## DISCLOSURE

*Approval of the research protocol*: The study protocol was approved by the Ethics Committee of the National Center for Global Health and Medicine, Japan. *Informed consent*: The J‐ECOH study was announced in each participating company through a poster; workers did not provide oral or written informed consent but were given the opportunity to opt for non‐participation in this study, in accordance with the Japanese Ethical Guidelines for Epidemiological Research. *Registry and the Registration number of the study/Trial*: N/A. *Animal Studies*: N/A. *Conflict of interest*: The authors have no conflicts of interest to declare.

## APPENDIX 1

Members of the Japan Epidemiology Collaboration on Occupational Health Study are: T. Mizoue, S. Akter, H. Hu, Y. Inoue, A. Fukunaga, I. Kashino, Z. Islam and M. Konishi, National Center for Global Health and Medicine, Tokyo, Japan; A. Nanri, Fukuoka Women's University, Fukuoka, K. Kurotani, National Institute of Health and Nutrition, Tokyo; K. Kuwahara, Teikyo University, Tokyo; T. Nakagawa, S. Yamamoto, T. Honda, and Y. Watanabe, Hitachi, Ltd., Ibaraki, Japan; S. Dohi and H. Okazaki, Mitsui Chemicals, Inc, Tokyo, Japan; T. Imai, Occupational Health Support Company, Tokyo; A. Nishihara, Azbil Corporation, Tokyo, Japan; N. Sasaki and T. Ogasawara, Mitsubishi Fuso Truck and Bus Corporation, Kanagawa, Japan; A. Uehara, Seijinkai Shizunai Hospital, Hokkaido; M. Yamamoto, YAMAHA CORPORATION, Shizuoka, Japan; T. Miyamoto, M. Hasegawa, and M. Shirozu, Nippon Steel & Sumitomo Metal Corporation Kimitsu Works, Chiba, Japan; I. Kabe, KUBOTA Corporation, Ibaraki, Japan; T. Kochi, and M. Eguchi, Furukawa Electric Co., Ltd., Tokyo, Japan; T. Murakami, C. Shimizu, M. Shimizu, N. Gonmori and A. Ogasawara, Mizue Medical Clinic, Keihin Occupational Health Center, Kanagawa, Japan; N. Kato and A. Tomizawa, Fuji Electric Co., Ltd., Kanagawa, Japan; K. Tomita, Healthplant., Tokyo, Japan; S. Nagahama, All Japan Labour Welfare Foundation, Tokyo, Japan; N. Kunugita, University of Occupational and Environmental Health, Fukuoka; T. Sone, National Institute of Public Health, Saitama, Japan; K. Fukasawa, ADVANTAGE Risk Management Co., Ltd., Tokyo, Japan; A. Hori, University of Tsukuba, Ibaraki, Japan; C. Nishiura, Tokyo Gas Co., Ltd., Tokyo, Japan; C. Kinugawa, Healc Corporation, Tokyo, Japan; R. Kuroda and K. Yamamoto, The University of Tokyo, Tokyo, Japan; M. Ohtsu, Himawari Industrial Physician & Occupational Health Consultant Office, Saitama, Japan; N. Sakamoto and Y. Osaki, Health Design Inc, Tokyo, Japan; T. Totsuzaki, Mizuho Health Insurance Society, Tokyo, Japan; M. Endo, Juntendo University, Tokyo, Japan; T. Itoh, New Japan Radio Co., Ltd., Tokyo, Japan; M. Kawashima, Central Japan Railway Company, Aichi, Japan; M. Masuda, AEON Co., Ltd., Chiba, Japan; K. Kitahara and T. Yokoya, Mitsubishi Hitachi Power Systems, Ltd., Kanagawa, Japan; K. Fukai, K. Odagami, and Y. Kobayashi, HOYA Corporation, Tokyo.
