# Supplementary material for: Five‐year cumulative incidence of overweight and obesity, and longitudinal change in body mass index in Japanese workers: The Japan Epidemiology Collaboration on Occupational Health Study
Source: J Occup Health. 2019 Nov 2;62(1):e12095. doi: 10.1002/1348-9585.12095 (PMC6970396; doi:10.1002/1348-9585.12095)
Supplement: Supplementary file 1 [file JOH2-62-e12095-s001.docx]

**Supplemental Table 1.** Hazard ratio (95% confidence Interval) of overweight and obesity according to baseline age groups (according to BMI cut-off point for Asian population).

| Age group (years) | Men | | |  | Women | | |
| --- | --- | --- | --- | --- | --- | --- | --- |
|  | no. of cases | Incidence rate (per 1000 person year) | HR (95% CI)^*^ |  | no. of cases | Incidence rate (per 1000 person year) | HR (95% CI)^*^ |
| **Overweight**^§^ |  |  |  |  |  |  |  |
| Total | 8032 | 66.9 |  |  | 1035 | 32.1 |  |
| 20-24 | 553 | 62.9 | 0.96 (0.86-1.06) |  | 76 | 33.0 | 1.05 (0.77-1.41) |
| 25-29 | 890 | 67.2 | 1.05 (0.95-1.15) |  | 103 | 27.1 | 0.87 (0.66-1.15) |
| 30-34 | 1003 | 66.4 | 1.02 (0.93-1.12) |  | 140 | 33.5 | 1.03 (0.79-1.34) |
| 35-39 | 1459 | 69.6 | 1.09 (0.99-1.18) |  | 240 | 32.0 | 1.04 (0.82-1.32) |
| 40-44 | 1291 | 69.5 | 1.10 (1.01-1.20) |  | 186 | 34.5 | 1.13 (0.88-1.44) |
| 45-49 | 1080 | 68.6 | 1.08 (0.99-1.18) |  | 124 | 31.2 | 1.00 (0.76-1.30) |
| 50-54 | 958 | 63.8 | 1.00 |  | 98 | 30.7 | 1.00 |
| 55-59 | 798 | 63.6 | 1.01 (0.92-1.11) |  | 68 | 35.9 | 1.23 (0.90-1.68) |
| p for trend |  |  | 0.71 |  |  |  | 0.22 |
| **Obesity**^¶^ |  |  |  |  |  |  |  |
| Total | 3522 | 14.2 |  |  | 344 | 7.4 |  |
| 20-24 | 206 | 15.7 | 1.21 (1.02-1.43) |  | 21 | 7.9 | 0.96 (0.55-1.65) |
| 25-29 | 336 | 15.2 | 1.22 (1.05-1.40) |  | 35 | 8.4 | 1.05 (0.65-1.69) |
| 30-34 | 385 | 13.8 | 1.07 (0.94-1.23) |  | 44 | 8.5 | 1.05 (0.67-1.66) |
| 35-39 | 681 | 15.7 | 1.27 (1.13-1.43) |  | 79 | 9.1 | 1.13 (0.75-1.70) |
| 40-44 | 633 | 15.4 | 1.25 (1.11-1.42) |  | 66 | 6.9 | 1.22 (0.80-1.86) |
| 45-49 | 489 | 13.5 | 1.10 (0.96-1.25) |  | 38 | 7.1 | 0.91 (0.57-1.46) |
| 50-54 | 438 | 12.3 | 1.00 |  | 33 | 9.6 | 1.00 |
| 55-59 | 354 | 12.4 | 1.03 (0.89-1.18) |  | 28 | 35.6 | 1.36 (0.82-2.25) |
| p for trend |  |  | 0.01 |  |  |  | 0.59 |

^§^Number of normal weight participants at baseline; ^¶^ Number of non-obese participants at baseline.

^*^Based on Cox proportional hazards analysis. Hazard ratio (HR) with 95% confidence interval (CI) was adjusted by worksite (11 work site) and smoking status (current or non-current).

**Supplemental Table 2.** Cumulative incidence of overweight and obesity according to age group during 5-year follow-up period in WHO BMI Classification

| Age group (years) | Men | |  | Women | |
| --- | --- | --- | --- | --- | --- |
|  | No. | Incidence, no. (%) |  | No. | Incidence, no. (%) |
| **Overweight §** | | | | | |
| Total | 34968 | 5418 (15.5) |  | 5829 | 471 (8.1) |
| 20-24 | 2164 | 335 (15.5) |  | 343 | 25 (7.3) |
| 25-29 | 3436 | 547 (15.9) |  | 464 | 25 (5.4) |
| 30-34 | 4210 | 638 (15.2) |  | 633 | 59 (9.3) |
| 35-39 | 6535 | 1091 (16.7) |  | 1415 | 104 (7.3) |
| 40-44 | 6031 | 991 (16.4) |  | 1160 | 110 (9.5) |
| 45-49 | 5194 | 814 (15.7) |  | 869 | 76 (8.7) |
| 50-54 | 4863 | 679 (14.0) |  | 664 | 48 (7.2) |
| 55-59 | 2535 | 323 (12.7) |  | 281 | 24 (8.5) |
| **Obesity ¶** | | | | | |
| Total | 46318 | 1359 (2.9) |  | 6580 | 154 (2.3) |
| 20-24 | 2483 | 82 (3.3) |  | 372 | 9 (2.4) |
| 25-29 | 4102 | 146 (3.6) |  | 499 | 14 (2.8) |
| 30-34 | 5320 | 164 (3.1) |  | 704 | 24 (3.4) |
| 35-39 | 8686 | 296 (3.4) |  | 1562 | 27 (1.7) |
| 40-44 | 8324 | 271 (3.3) |  | 1306 | 25 (1.9) |
| 45-49 | 7192 | 187 (2.6) |  | 1000 | 26 (2.6) |
| 50-54 | 6709 | 150 (2.2) |  | 778 | 18 (2.3) |
| 55-59 | 3502 | 63 (1.8) |  | 359 | 11 (3.1) |

WHO BMI classification: overweight: 25.0 kg/m^2^≤BMI<30.0 kg/m^2^, obesity: ≥30.0 kg/m^2^.

§Number of normal weight participants at baseline; ¶ Number of non-obese participants at baseline.

**Supplemental Table 3.** Hazard ratio (95% confidence interval) of overweight and obesity according to baseline age groups among individuals who were overweight at baseline (according to BMI cut-off point for Asian population).

| **Age group (years)** | Men | | |  | Women | | |
| --- | --- | --- | --- | --- | --- | --- | --- |
|  | no. of cases | Incidence rate (per 1000 person year) | HR (95% CI)^*^ |  | no. of cases | Incidence rate (per 1000 person year) | HR (95% CI)^*^ |
| Total | 3462 | 31.8 |  |  | 331 | 41.8 |  |
| 20-24 | 196 | 61.7 | 2.45 (2.07-2.91) |  | 19 | 49.4 | 1.69 (0.95-3.01) |
| 25-29 | 317 | 46.6 | 1.88 (1.63-2.17) |  | 32 | 65.5 | 2.50 (1.52-4.10) |
| 30-34 | 380 | 36.7 | 1.46 (1.27-1.67) |  | 42 | 52.1 | 1.93 (1.21-3.08) |
| 35-39 | 671 | 36 | 1.49 (1.32-1.68) |  | 77 | 54.8 | 2.05 (1.35-3.10) |
| 40-44 | 625 | 32.2 | 1.34 (1.18-1.51) |  | 64 | 43.6 | 1.62 (1.05-2.47) |
| 45-49 | 485 | 27.3 | 1.14 (1.00-1.29) |  | 37 | 30.2 | 1.14 (0.71-1.83) |
| 50-54 | 436 | 23.9 | 1.00 |  | 32 | 26.1 | 1.00 |
| 55-59 | 352 | 24.2 | 1.04 (0.90-1.20) |  | 28 | 30.1 | 1.22 (0.73-2.02) |
| p for trend |  |  | <0.01 |  |  |  | <0.01 |

^*^Based on Cox proportional hazards analysis. Hazard ratio (HR) with 95% confidence interval (CI) was adjusted by worksite (11 work site) and smoking status (current or non-current).
